# Recent and speculative remote myocardial trauma: a case series

**DOI:** 10.1093/ehjcr/ytae349

**Published:** 2024-07-12

**Authors:** Zhijuan Lu, Xiaocong Zhang, Yanling Huang, Jia Song, Chaoqun Zhang, Qiang Wang, Handong Wu, Xinsheng Huang

**Affiliations:** Department of Ultrasound, Dongguan Hospital of Guangzhou University of Chinese Medicine, No. 22 Songshan Lake Road, Dongguan 523000, China; Department of Cardiology, Foshan Fosun Chancheng Hospital, No. 3 Sanyou South Road, Foshan 528000, China; Department of Cardiology, Jinshazhou Hospital of Guangzhou University of Chinese Medicine, No. 1 Lichuan East Street, Guangzhou 510168, China; Department of Ultrasound, Dongguan Hospital of Guangzhou University of Chinese Medicine, No. 22 Songshan Lake Road, Dongguan 523000, China; Department of Cardiology, Foshan Fosun Chancheng Hospital, No. 3 Sanyou South Road, Foshan 528000, China; Department of Cardiology, Foshan Fosun Chancheng Hospital, No. 3 Sanyou South Road, Foshan 528000, China; Department of Cardiology, Jinshazhou Hospital of Guangzhou University of Chinese Medicine, No. 1 Lichuan East Street, Guangzhou 510168, China; Department of Cardiology, Jinshazhou Hospital of Guangzhou University of Chinese Medicine, No. 1 Lichuan East Street, Guangzhou 510168, China

**Keywords:** Myocardial trauma, Dissecting tear, Rupture, Cardiac imaging, Case series

## Abstract

**Background:**

Cardiac blunt trauma clinically presents as a spectrum of injuries of varying severity. However, the diagnosis of complications of remote myocardial trauma is often challenging, especially if the patient forgets to mention a remote history of chest trauma.

**Case summary:**

In this study, we present a patient who recently experienced traumatic myocardial dissection and interventricular septal rupture, alongside three patients exhibiting a mimic double-chambered left ventricle, indicative of prior remote myocardial trauma potentially associated with myocardial dissecting tear.

**Discussion:**

Patients with recent severe myocardial injury are detectable through cardiac imaging. However, forgotten remote myocardial trauma can lead to adverse myocardial remodelling, heart failure, and arrhythmias. Long-term myocardial remodelling can obscure initial myocardial imaging characteristics, posing challenges in interpretation. Our case series suggests that remote myocardial trauma may be more prevalent than commonly thought of in clinical practice.

**Learning points**

- An abnormal morphological left ventricle is often interpreted as ventricular aneurysm, pseudoaneurysm, diverticulum, double-chambered left ventricle, or dilated cardiomyopathy in cardiac imaging.
- The case series reports that provides evidence that some patients with a mimic double-chamber left ventricle of cardiac imaging may experience myocardial dissecting laceration and rupture into the left ventricle because of remote blunt cardiac trauma observed in clinical practice. A remote cardiac trauma may be more prevalent than commonly thought of in clinical practice.

## Introduction

Blunt cardiac trauma can arise from various scenarios, such as traffic accidents, sports activities, falls, and thoracic wall crush injuries, sometimes resulting in sudden death with or without obvious structural cardiac traumas.^1,2^ Diagnostic methods include serum troponin levels, electrocardiography, echocardiography, and computed tomography (CT), but no single test offers high specificity and sensitivity, and consensus remains elusive. While relatively common, blunt cardiac trauma often leads to minimal injuries and slight morbidity,^3^ with some patients not seeking medical attention for mild symptoms. However, complications from remote blunt cardiac trauma may prompt medical intervention.^4^ In this paper, we present clinical and imaging data on adult subjects, highlighting a patient with recent traumatic myocardial dissection and interventricular septal rupture, and three patients with suspected remote myocardial trauma, exhibiting a mimic double-chambered left ventricular morphology possibly associated with myocardial dissecting tear and rupture into the left ventricle. This case series underscores the importance of comprehensive history taking and awareness of complications from remote blunt cardiac trauma to avoid unnecessary and potentially risky interventions.

## Summary figure

**Table:** 

Case 1	3 September 2010	Sustained multiple injuries in a motor vehicle accident
	6 September 2010	Echo suggestive of an interventricular septal myocardial dissecting laceration and rupture with a small left-to-right shunt
	14 September 2010	These cardiac injuries were successfully repaired by a medical team and the patient was subsequently discharged
	October 2011	Echo suggestive of mild reductions in the contractility of the septum and apex, a normal left ventricular ejection fraction, mild tricuspid valvular regurgitation, and no residual ventricular-level shunt. Persistent anterior T-wave inversion and 3.8% of monomorphic premature ventricular contractions (PVCs), then loss of follow-up
Case 2	12 January 2019	Demonstrated an abnormal electrocardiogram
	10 December 2019	Normal coronary angiography. Left ventricular angiography with a mimic double-chambered left ventricle
	13 January 2023	Echo suggestive of a myocardial dissecting-like tear in the interventricular septal and anterior left ventricular wall and apical regions and a rupture into the left ventricle. A valve-like movement flap between the two cavities with mildly reduced interventricular septal and apical contractility. Upon detailed questioning, the patient recalled that he had fallen from a tall tree 30 years ago, experiencing severe crushing chest pain
	13 January 2023	Cardiac magnetic resonance suggestive of a mimic double-chambered left ventricle
	January 2024	Remains asymptomatic
Case 3	31 August 2023	A 40-year recurrent unexplained syncope. Holter monitoring confirming sinus rhythm and incidental atrial contractions and PVCs
	6 September 2023	Echo suggestive of a myocardial dissecting-like tear in the interventricular septal region and anterior left ventricular wall that communicated with the left ventricle. Upon detailed questioning, the patient recalled a remote blunt chest trauma incident 46 years ago
	7 September 2023	Cardiac magnetic resonance suggestive of myocardial dissection and late gadolinium enhancement at the basal anteroseptal segment in a non-ischaemic pattern
	8 September 2023	Coronary angiography ruling out significant coronary obstruction
	February 2024	Remains well, with no further episodes of syncope
Case 4	30 April 2023	Sought medical assistance for unexplained nocturnal incontinence
	15 June 2023	Coronary angiography suggestive of a right dominant coronary circulation without significant coronary obstruction
	18 July 2023	Holter monitoring suggestive of 877 monomorphic PVCs, neither couplet nor non-sustained ventricular tachycardia
	22 September 2023	Echo suggestive of a mimic double-chambered left ventricle with reduced contractility in the interventricular septal and apical regions. Upon detailed questioning, the patient recalled a remote blunt chest trauma incident 8 years ago
	February 2024	Remains well, with no further episodes of nocturnal incontinence

## Case presentation

### Case 1

**Figure 1:**
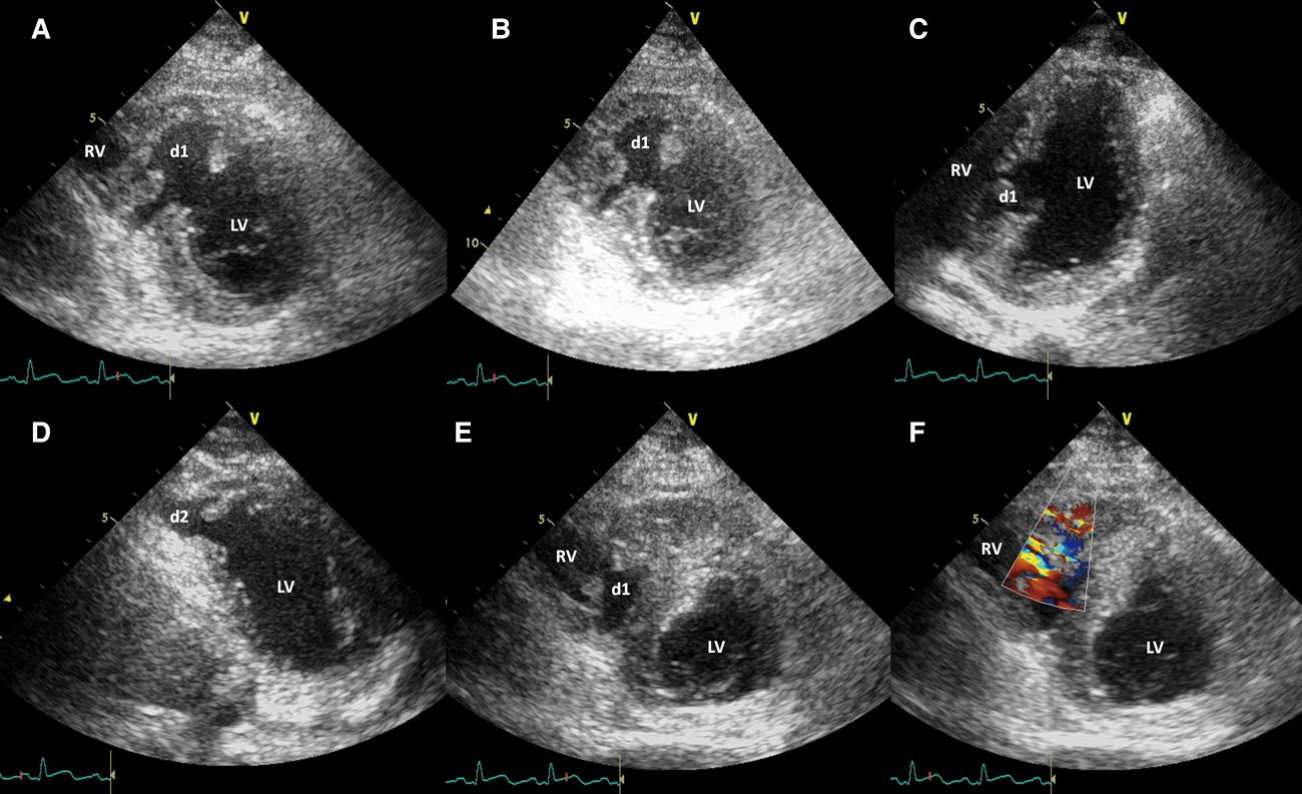
Echocardiography demonstrating an interventricular septal myocardial dissecting tear (d1) and rupture into the left and right ventricles (*A–C*) and an apical myocardial dissecting laceration (d2) and rupture into the left ventricle (*D*). An interventricular septal dissecting rupture with a small left-to-right shunt is demonstrated (*E* and *F*). LV, left ventricle; RA, right atrium; RV, right ventricle.

A 29-year-old man sustained multiple injuries in a motor vehicle accident 3 days prior, including a brief loss of consciousness because of brain trauma, thoracic trauma resulting in bilateral pleural effusion, and a right knee fracture. Upon admission, he remained conscious and in a stable haemodynamic condition. There was a Grade 3/6 holosystolic murmur over the precordium. Echocardiography revealed an interventricular septal myocardial dissecting laceration and rupture with a small left-to-right shunt, tricuspid valve chordal rupture, reduced contractility of the septum and apex, and a 69% left ventricular ejection fraction (LVEF; *Figure 1* and Supplementary material online, *Video S1*). Coronary angiography did not reveal significant coronary disease or dissection. On the 8th day after admission, the medical team successfully repaired these cardiac injuries and subsequently discharged him. During the 1-year follow-up, the patient remained asymptomatic, without experiencing dizziness or syncope. Echocardiography showed mild reductions in the contractility of the septum and apex, a normal LVEF, mild tricuspid valvular regurgitation, and no residual ventricular-level shunt. The electrocardiogram displayed persistent anterior T-wave inversion. Holter monitoring recorded 3.8% of monomorphic premature ventricular contractions (PVCs), without couplets or non-sustained ventricular tachycardia. Afterwards, the patient was lost to follow-up.

### Case 2

**Figure 2:**
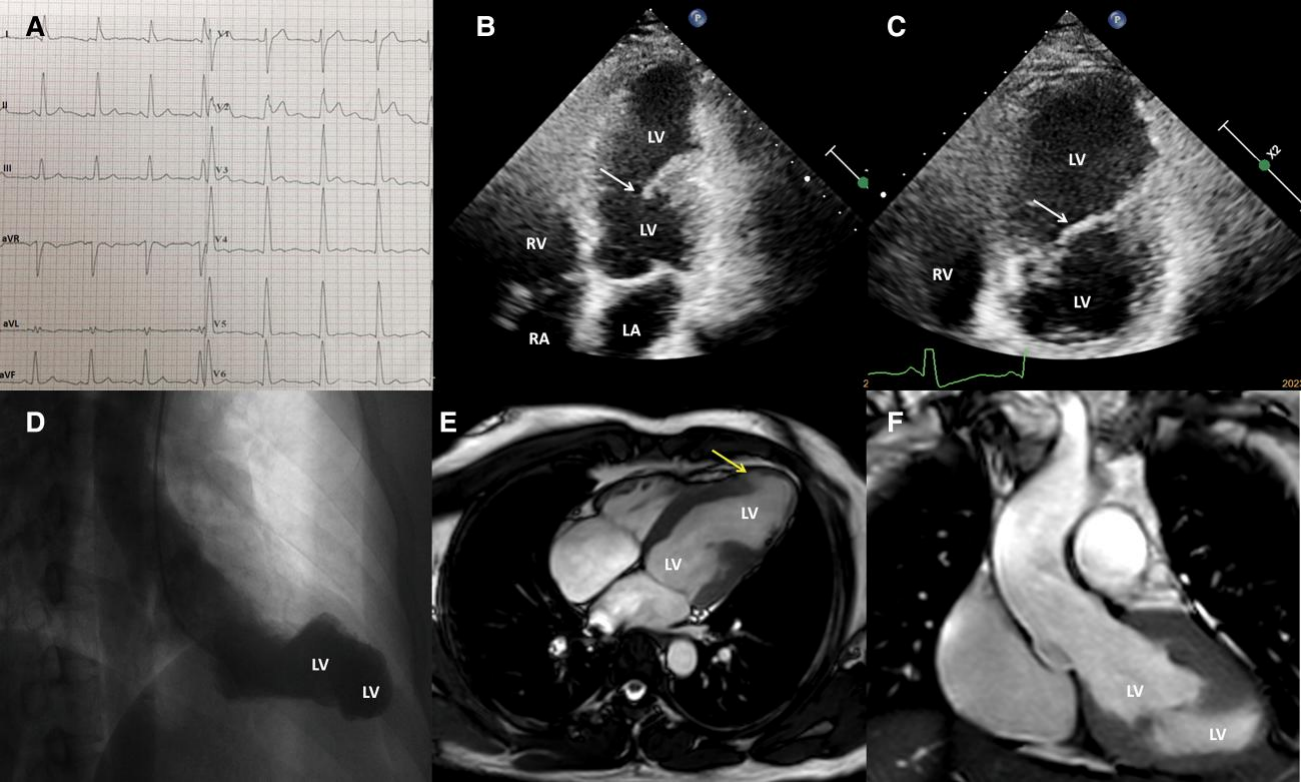
Electrocardiography reveals non-specific intraventricular conduction delay and ST-segment elevation in V2 (*A*). Echocardiography reveals a myocardial dissecting-like tear in the interventricular septal and anterior left ventricular wall and apical regions and rupture into the left ventricle, as well as a valve-like swinging flap (arrow) between the two cavities (*B* and *C*). Left ventricular angiography (*D*) and cardiac magnetic resonance (*E* and *F*) reveal a mimic double-chambered left ventricle. A small localized bulging of myocardial jagged laceration is revealed (arrow) (*E*). LA, left atrium; LV, left ventricle; RA, right atrium; RV, right ventricle.

An asymptomatic 41-year-old man underwent cardiac evaluation because of an abnormal electrocardiogram (*Figure 2*). The results of other examinations were normal. Coronary angiography confirmed normal findings. Left ventricular angiography and cardiac magnetic resonance imaging revealed a mimic double-chambered left ventricle (*Figure 2*). Echocardiography revealed a myocardial dissecting-like tear in the interventricular septal and anterior left ventricular wall and apical regions and a rupture into the left ventricle. There was a valve-like movement flap between the two cavities with mildly reduced interventricular septal and apical contractility as well as a 49% LVEF (*Figure 2* and Supplementary material online, *Video S2*). Holter monitoring recorded 21 monomorphic PVCs and 12 monomorphic premature supraventricular contractions, including 1 triplet. Upon detailed questioning, the patient recalled a remote blunt chest trauma incident. At the age of 11 years, he had fallen ∼5 m from a tall tree while attempting to retrieve bird eggs, experiencing severe crushing chest pain and a prolonged recovery time. Remarkably, he did not seek medical attention. His chest pain spontaneously resolved after a month. Therefore, we attribute the left ventricular remodelling to an interventricular septal and apical myocardial dissecting laceration and rupture into the left ventricle, resulting from the remote cardiac contusion. The patient remained asymptomatic at the 5-year follow-up.

### Case 3

**Figure 3:**
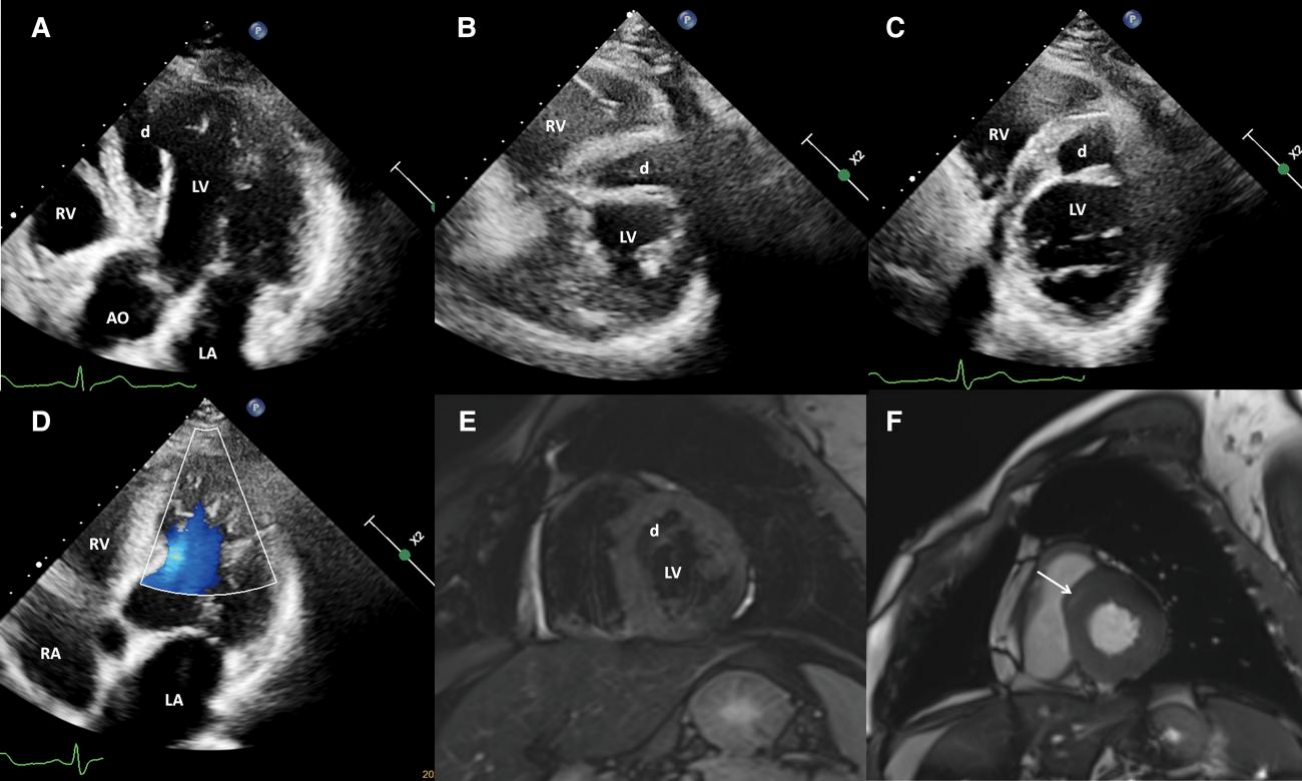
Echocardiography reveals a myocardial dissecting-like tear and rupture into the left ventricle in the interventricular septal and anterior left ventricular wall and apical regions (d) without haemodynamic obstruction between the left ventricular and the dissecting cavities (*A–C*) with mild reduced interventricular septal and apical contractility. There is a valve-like movement flap (*A*) between the left ventricle and the dissecting cavity without haemodynamic obstruction (*D*). Cardiac magnetic resonance reveals the myocardial dissection (d) (*E*) and the late gadolinium enhancement (white arrow) at the basal anteroseptal segment (*F*). AO, aorta; LA, left atrium; LV, left ventricle; RA, right atrium; RV, right ventricle.

A 53-year-old man with a 40-year history of recurrent unexplained syncope had another attack a week ago, and he was referred to our hospital for further evaluation. On admission, his heart rate and blood pressure were 66 b.p.m. and 118/80 mmHg, respectively. The results of other physical examinations were also normal. The electrocardiogram displayed a sinus rhythm and an incomplete right bundle branch block. Computed chest, abdomen/pelvis tomography, and brain magnetic resonance imaging revealed unremarkable findings. Holter monitoring confirmed sinus rhythm and incidentally detected atrial contractions and PVCs. Coronary angiography ruled out significant coronary obstruction. Echocardiography revealed a myocardial dissecting-like tear in the interventricular septal and anterior left ventricular wall that communicated with the left ventricle (*Figure 3*). There was a valve-like movement flap without haemodynamic obstruction between the left ventricle and the dissecting cavity (*Figure 3* and Supplementary material online, *Video S3*). There was mildly reduced contractility in the interventricular septal and apical regions with a 48% LVEF and mild tricuspid regurgitation with normal estimated pulmonary systolic pressure. Cardiac magnetic resonance also demonstrated the myocardial dissection and late gadolinium enhancement at the basal anteroseptal segment in a non-ischaemic pattern (*Figure 3*). Upon detailed questioning, the patient recalled a remote blunt chest trauma incident. When he was 7 years old, someone playfully tossed him into a haystack, resulting in a fall from a height of ∼2 m. He vividly remembered experiencing severe crushing chest pain with diaphoresis and dyspnoea. Importantly, he did not seek medical assistance, and his chest pain spontaneously resolved ∼1 month after the blunt chest trauma. A recurrent unexplained syncope had troubled him since he was 13 years old. As such, we attributed the ventricular remodelling to the interventricular septal and apical myocardial dissecting tear and rupture into the left ventricle because of the remote myocardial contusion. We speculated that severe ventricular arrhythmia was the cause of the syncope, despite Holter monitoring not capturing any serious arrhythmias. Nevertheless, the patient declined an implantable cardioverter defibrillator despite providing informed consent. Bisoprolol and sacubitril/valsartan therapies were administered to the patient. The patient remained well and did not present with syncope during the 6-month follow-up.

### Case 4

**Figure 4:**
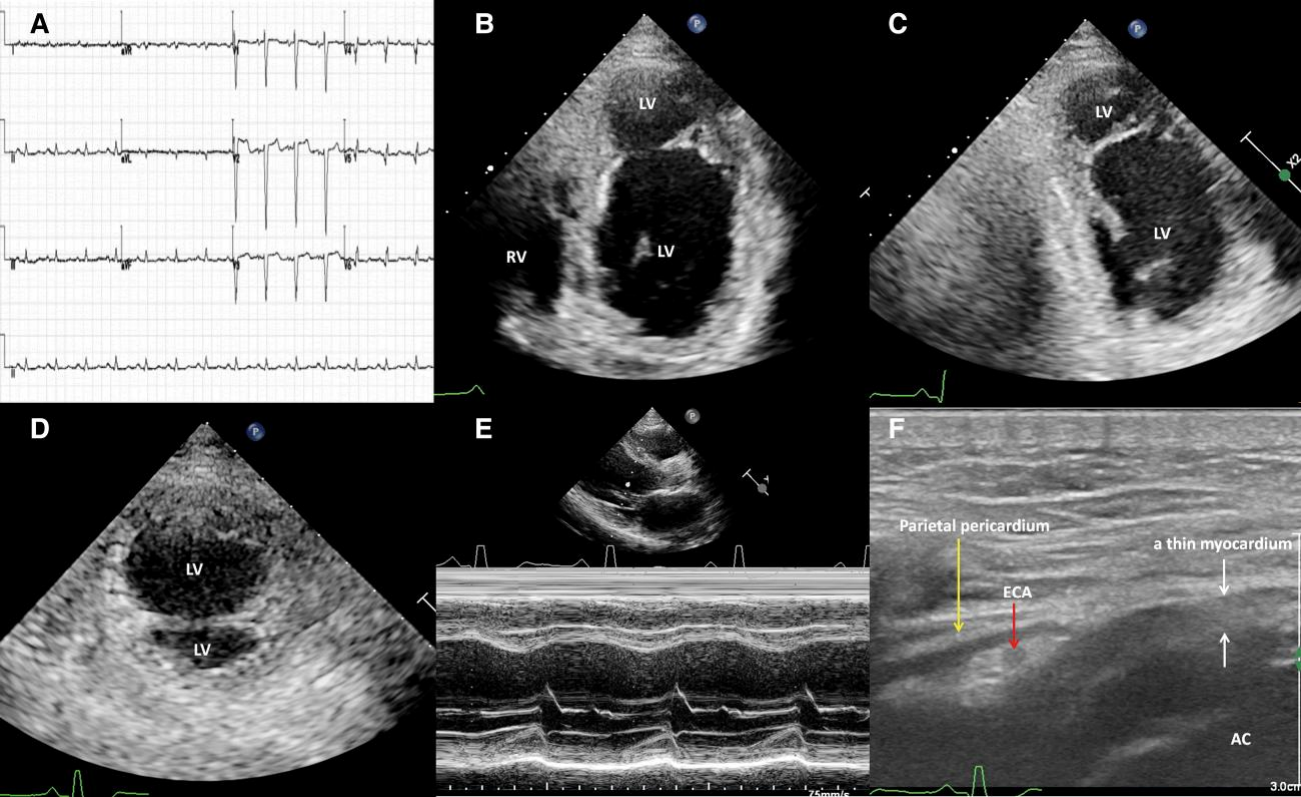
An electrocardiogram shows normal sinus rhythm, low voltage in limb leads, rS pattern in precordial Leads V1–V4, rs pattern in precordial Leads V5 and V6, and non-specific ST–T abnormalities (*A*). Echocardiography reveals a mimic double-chambered left ventricle with reduced contractility in the interventricular septal and apical regions in the apical four-chamber (*B*) and two-chamber (*C*) and short-axial (*D*) views. An M-mode shows the basal septum and left ventricular posterior wall with near normal contractility (*E*). A high-frequency ultrasound shows the apical thin myocardium with hypokinesia (*F*). AC, accessory chamber; ECA, epicardial coronary artery; LV, left ventricle; RV, right ventricle.

A 45-year-old man sought medical assistance for unexplained nocturnal incontinence, without manifest symptoms of heart failure. His physical examination results were normal. Brain magnetic resonance imaging yielded unremarkable results. His electrocardiogram demonstrated a normal sinus rhythm and low voltage in limb leads, an rS pattern in precordial Leads V1–V4, an rs pattern in precordial Leads V5 and V6, and non-specific ST–T wave abnormalities (*Figure 4*). Echocardiography unveiled a mimic double-chambered left ventricle with reduced contractility in the interventricular septal and apical regions (*Figure 4*) and a 34% LVEF. A high-frequency ultrasound showed a thin apical myocardium with hypokinesia (*Figure 4* and Supplementary material online, *Video S4*). There was no haemodynamic obstruction between the two left ventricular cavities. The serum myocardial enzymes showed normal levels, and there was a slight elevation in troponin T. Coronary angiography showed a right dominant coronary circulation without significant coronary obstruction. The mid-segment of his left anterior descending artery had a focal 30% stenosis with thrombolysis in myocardial infarction (TIMI) flow Grade 3. Holter monitoring revealed 877 monomorphic PVCs, with neither couplet nor non-sustained ventricular tachycardia.

Upon detailed questioning, the patient recalled a remote blunt chest trauma incident 8 years ago. He was sent to the local hospital, and he stayed in the local emergency department for 24 h. A physical examination showed slight chest wall abrasions. A thoracic CT did not show any abnormality. However, he did not undergo a detailed cardiac evaluation. His chest pain spontaneously resolved ∼1 month after the blunt chest trauma. Therefore, we attributed the mimic double-chambered left ventricle to the interventricular septal and apical myocardial dissecting tear and rupture into the left ventricle because of the remote cardiac contusion. We speculated that severe ventricular arrhythmia caused the patient’s symptoms, although Holter monitoring did not record any serious arrhythmias. The patient declined an implantable cardioverter defibrillator despite providing informed consent. Bisoprolol and sacubitril/valsartan therapies were administered to the patient. The patient remained well and did not present with nocturnal incontinence at the 10-month follow-up.

## Discussion

Cardiac blunt trauma clinically presents as a spectrum of injuries of varying severity. Medical professionals can easily detect patients with recent severe myocardial injury. Although most patients with blunt chest trauma have chest wall lesions, the absence of thoracic lesions decreases the suspicion but never excludes cardiac injury. Recent cardiac blunts may produce only mild symptoms, such as palpitations or precordial pain, which is often attributed to concomitant musculoskeletal injury.^5^ Patients with mild symptoms may not seek medical attention. Only for these reasons, it is difficult to diagnose the complications of remote cardiac blunt trauma, especially if the patient forgets to mention a remote history of chest trauma. Therefore, such patient cases indicate that, while encountering an unexplained abnormal morphological left ventricle, especially a mimic double-chambered left ventricle, patients should be carefully inquired about remote blunt chest trauma.

Blunt thoracic trauma can induce myocardial lesions by several mechanisms, including direct transfer of kinetic energy during the impact on the chest, a sudden forceful deceleration process of the heart, and compression of the heart between the sternum and the spine.^2^ Interventricular septal rupture following blunt chest trauma seems to occur because of either a laceration or a contusion and subsequent necrosis. Traumatic coronary artery injury and dissection is a potentially life-threatening complication to consider in patients presenting with prolonged chest pain following chest trauma.^4^ In our case series, we consider the mechanism of rupture to be direct laceration rather than necrosis, because there is no evident coronary artery obstruction or dissection. The accessory chamber shape of the lesion, myocardial jagged laceration, and a valve-like swinging flap. The accessory chamber shape of the lesion, myocardial jagged laceration and a valve-like swinging flap all support the rupture of myocardial dissection. The close anatomical proximity of the apical region to the anterior chest wall likely renders it more susceptible to direct trauma-related damage.

In the classic cardiac rupture presentation of cardiac rupture, myocardial laceration causes blood to pass through the full thickness of the myocardium. However, there are instances where the rupture is non-transmural, forming a blood-filled intra-myocardial cavity that is externally constrained by segments of the myocardium and the pericardial layer.^6^ Our case series indicates that patients with non-fatal cardiac contusion may have long-term survival with or without symptoms. Cardiac arrhythmia, both in clinical cases and in experimental animals, is one of the most frequent signs of recent myocardial contusion.^3^ Ventricular arrhythmias and cardiac failure are the most important complications of remote blunt cardiac trauma in our case series.

We observe a viable myocardium along the right and left aspects of the ventricular septum in our Case 1 patient with recent myocardial dissecting tear and rupture. The echocardiography easily detected the myocardial dissecting tear and rupture. The tearing viable myocardium maintained the original myocardial echocardiographic features. However, varying thicknesses of a viable or non-viable myocardium are seen along the endocardial and epicardial aspects of remote myocardial dissecting tear and rupture. The most vulnerable myocardial layer may rupture. The sub-endocardial region is the most vulnerable part of the ventricular wall, whereas the sub-epicardial myocardium tends more to remain viable.^7^ Similar to the case following a myocardial infarction, adverse myocardial remodelling following remote blunt cardiac trauma forms the structural foundation for causing alterations in cardiac morphology, contributing to heart failure and arrhythmias. Cardiac repair post-cardiac trauma initiates sterile inflammation and infiltration of immune cells, facilitating the removal of damaged cells and extracellular matrix tissue. This is succeeded by fibroblast proliferation and the formation of scar tissue.^8^ The loss of viable myocardial tearing flaps cannot help maintain original myocardial imaging features because of long-term myocardial remodelling, which makes interpreting pathology potentially difficult in clinical practice.

The congenital double-chambered left ventricle is an exceedingly rare anomaly. It has predominantly been reported in children and is associated with cardiac systolic dysfunction and other cardiac abnormalities.^3,4,9,10^ The current cardiac imaging modality indicates an increasing frequency of acquired morphological double-chambered left ventricles in adult populations.^11,12^ The aetiology of the congenital double-chambered left ventricle is unknown, but the acquired mimic double-chambered left ventricle may be caused by myocardial remodelling following myocardial injury. In our case series, we observed that cardiac imaging of the mimic double-chambered left ventricle also significantly differs from the morphology of the classic congenital double-chambered left ventricle.^9,10^ Myocardial dissecting laceration and rupture into the left ventricle, or intra-myocardial dissecting haematoma, are typically considered rare complications associated with myocardial infarction, chest trauma, or medical procedures.^6,13–16^ Therefore, we speculate that some patients with a mimic double-chambered left ventricle of cardiac imaging may experience myocardial dissecting laceration and rupture into the left ventricle because of remote blunt cardiac trauma observed in clinical practice.

To the best of our knowledge, there is limited literature dedicated to remote cardiac trauma. Our case series underscores the significance of meticulous history taking and the need to consider the complications stemming from remote cardiac blunt trauma, thereby averting unnecessary and potentially hazardous interventions. However, the limitation of this case series is that direct evidence of remote myocardial trauma has not yet been found, and more careful consideration should be given to the association between a mimic double-chambered left ventricle and a remote myocardial trauma.

## Supplementary Material

ytae349_Supplementary_Data

## Data Availability

The original contributions presented in the study are included in the article and the Supplementary material, and further inquiries can be directed to the corresponding author.

## Lead author biography

Dr Zhijuan Lu is a graduate of Guangdong Medical University, China, who is pursuing a career in cardiovascular imaging.

## Notes

**Consent:** The authors confirm that written consent for submission and publication of the case series, including images and associated text, has been obtained from the patients in line with COPE guidelines.

**Funding:** This work was supported by the 14th Five-Year Medical Key Specialist Construction Project of Foshan (grant no. FSZD145024 to Q.W.).
